# Increasing Nature Connection in Children: A Mini Review of Interventions

**DOI:** 10.3389/fpsyg.2020.00492

**Published:** 2020-03-19

**Authors:** Alexia Barrable, David Booth

**Affiliations:** ^1^School of Education and Social Work, University of Dundee, Dundee, United Kingdom; ^2^School of Life Sciences, University of Dundee, Dundee, United Kingdom

**Keywords:** nature connection, children, intervention, environmental education, sustainability

## Abstract

Half of the world’s population live in the urban environment. Lifestyle changes in the 20th century have led to spending more time indoors and less in nature. Due to safety concerns, longer hours in formal education, as well as lack of suitable outdoor environments, children in particular have been found to spend very little time outdoors. We have an opportunity, both timely and unique to have our children (re)connect with nature. Nature connection is a subjective state and trait that encompasses affective, cognitive, and experiential aspects in addition to being positively associated with wellbeing, and strong predictor of pro-environmental attitudes and behaviors. This mini-review brings together recent studies that report on interventions to increase nature connection in children. Fourteen studies were identified through electronic searches of Web of Science, Scopus, PsychInfo, ERIC, and Google Scholar. The review aims to offer an overview of the interventions identified, provide a snapshot of the current state of the literature, briefly present themes and trends in the studies identified in relation to nature connection in young people, and propose potential guidelines for future work.

## Introduction

In the 21st century, numerous voices have been calling for children and adults to (re)connect with nature, both as a wellbeing intervention for humans, but also for environmental sustainability (Miller, 2006; Barker, 2007; Louv, 2008; Capaldi et al., 2015; Díaz et al., 2015). Nature connection, the concept that describes the human–nature relationship, has been described in numerous ways. These related, but not identical constructs have at different times been defined as inclusion of nature in self (Schultz, 2002), nature relatedness (Nisbet et al., 2009), emotional affinity toward nature (Müller et al., 2009), and nature connectedness (Mayer and Frantz, 2004). Despite the subtle differences in these constructs, as well as different instruments to measure them, the underlying construct is very similar and it refers to our perceived and subjective connection to the non- human natural world (Capaldi et al., 2014). A review exploring the similarities and differences between the constructs and measures found that not only do the measures correlated strongly with each other, but that they also shared similar correlations with measures of wellbeing, and ecological beliefs and behaviors (Tam, 2013). For this reason, this paper will include all the constructs mentioned above, and use the umbrella term “nature connection” for ease.

Several studies have found nature connection is positively associated with wellbeing in adults and children (Mayer and Frantz, 2004; Howell et al., 2011; Nisbet and Zelenski, 2013; Capaldi et al., 2014; Zelenski and Nisbet, 2014; RSPB, 2015). Moreover, feeling close to the natural world has been found to correlate positively with pro-environmental attitudes and ecological behaviors (Mayer and Frantz, 2004; Leary et al., 2008; Nisbet et al., 2009; Frantz and Mayer, 2014). In fact, nature connectedness is a stronger predictor of ecological behaviors in children, than environmental knowledge (Otto and Pensini, 2017). For these reasons, nature connection has been identified as a suitable focus for assessing environmental education (EE) programs (Frantz and Mayer, 2014), as well as a distinct goal for early years’ environmental and outdoor education (Otto and Pensini, 2017; Barrable and Arvanitis, 2018; Barrable, 2019a, b).

Childhood is often seen as a time of development for values and beliefs (Wigfield and Eccles, 2002). There is also evidence to suggest that adult nature connection and environmental stewardship may have their roots in childhood (Wells and Lekies, 2006; Andrejewski et al., 2011). Therefore, this current mini-review focuses on activities and interventions that aim to promote nature connection in children. More specifically, the review aims to identify and summarize the key points of interventions that promote a connection to nature in people <18 years of age, and provide some guidelines for future research.

## Materials and Methods

### Inclusion Criteria

In order to find interventions that promote nature connection the author conducted a literature search adopting the following inclusion criteria. The articles identified had to (i) be published in peer-reviewed journals; (ii) be in the English language; (iii) have used experimental or quasi-experimental design, including randomized controlled trials (RCTs), pre- and post-testing with or without control groups, and included both between- and within-subjects testing; (iv) have nature connection as a dependent variable; (v) have used a validated instrument for that age group to measure nature connection; and finally (vi) majority of participants were under the age of 18 years.

### Data Sources and Search Strategy

In order to gain a comprehensive coverage of the literature, the following three-fold strategy was used.

(1) Keyword searches were undertaken in the following scientific databases: Web of Science, Scopus, PsychInfo, ERIC, and Google Scholar. The terms used were “nature relatedness,” “connection to nature,” and “nature connect^∗^,” in combination with “intervention,” “measure,” and “testing.”
(2) Specific appropriate journals (such as Journal of Environmental Psychology, Environment and Behavior, Ecopsychology, and others) were targeted and searched using the same terms as above.
(3) Finally, by using Google Scholar the first author manually looked through all publications that cited any of the articles of validation of nature connection measures.

The following information was extracted from each of the publications: age and number of participants, length and type of intervention, design, nature connection measure used, and finally effect size, if reported.

## Results

A total of 3794 articles were initially identified, with 635 remained after duplicates were removed. Those were then screened by title and abstract. Forty-three full articles were read and finally 14 articles were identified as meeting all inclusion criteria.

The ages of participants in the studies ranged from 6 years of age (Bruni et al., 2017) to 19 (Sellmann and Bogner, 2013). All of the studies included pre- and post-intervention measurements, while five also included a control group. The length of activities reported on varied widely, from a short, two-hour field trip reported in Boeve-de Pauw et al. (2019) to programs that lasted several weeks and included regular weekly classes (e.g., Hignett et al., 2018). Environments were also diverse, ranging from the South African bush to the Scottish Highlands, and included urban and wild nature, indoor environments, and coastal areas. Nine of the studies describe activities that were characterized by the authors as EE, while the rest were a mixture of outdoor leisure activities, camps, expeditions, and other educational activities. Several scales were used, which are reported in Table 1.

**TABLE 1:** Interventions to increase nature connection as identified in review.

Article	Age of participants (years)	Length of intervention	Type of intervention	Type of environment	Design	Control	Number of participants	Instrument used	Effect size (Cohen’s *d*)
Barton et al., 2016	11–18	5–11 days	Wildlife expeditions	Bush/highlands	Pre–post	No	130	CNS	≈0.96
Boeve-de Pauw et al., 2019	10–11	2 h	Field trip (EE)	Heathland	Pre–post	No	560	INS	≈0.26
Braun and Dierkes, 2017	7–18	1-day, 5-day	1-day field trip 5-day residential (EE)	Rainforest	Pre–post	Yes	601	INS	≈0.21
Bruni et al., 2017	6–16	Varied (30 days – activity 1 to 30–45 min)	*Get to know program* three studies for three activities (1) The Creative Arts Contest, (2) the Natural Treasure Adventure, and (3)Virtual Hikes	Urban nature	Pre–post	No	(1) 168 (2) 35 (3) 50	IAT nature (FlexiTwins)	≈0.37
Bruni et al., 2018	6–15	Day visit	Visit to natural history museum	Museum	Pre–post	No	238 (across two locations)	IAT nature (FlexiT wins)	≈0.15
Collado et al., 2013	Approx. 7–15	1–2 weeks	Summer camps	Mountain camp	Pre–post	Yes (urban camp)	397 (four different camps)	EAN	≈0.89
Ernst and Theimer, 2011	8–14	Seven different programs all which included sustained contact with nature	EE programs	Urban nature	Pre–post	Yes	Total 385	CNI	0
Hignett et al., 2018	13–16	12 weekly lessons	Surfing and EE program for “at risk” youth	Coast	Pre–post	No	58	INS	0
Kossack and Bogner, 2012	Approx. 10–16	1 day	Indoor and outdoor EE program	Woodland	Pre–post and follow up	Yes	123 (and 116 control) = 239	INS	≈0.42–0.71
Liefländer et al., 2013	9–13	4-days	EE program on water	Woodland	Pre–post and follow up	Yes	264	INS	≈0.3–0.65
Mullenbach et al., 2018	10–11	4-day	Residential outdoor EE program	Urban nature	Pre–post	Yes	163	Adapted CNS	≈0.11–0.25
San Jose and Nelson, 2017	9–11	4-day	4-day outdoor program	Woodland	Pre- and post and follow up	No	177	CNI	≈0.53
Schneider and Schaal, 2017	Approx. 10–16	1-day 5-day	EE program with use of geogames/treasure hunt game	Woodland	Pre–post	No	339	INS (and DCN)	≈0.2
Sellmann and Bogner, 2013	15–19	1-day	EE program	Urban nature	Pre–post, and follow up	Yes	114	INS	≈0.77
									

## Summary of Key Themes

### Participant Age as an Influencing Factor

Some studies looked at the effect of age and reported significant findings. Braun and Dierkes (2017) found that there were significant age-based differences between the samples tested for baseline nature connection, with younger children (10–12) having higher nature connection compared to the older (13–15) group. During analysis, for the 5-day programs 7–9-year olds exhibited the largest shift, while for the 1-day intervention, it was the 17–19-year-old group that showed the greatest positive shift. Finally, looking at follow up after 6 weeks, these two groups (10–12 and 17–19) exhibited highest retention of nature connection, with 13–15 showing the biggest decline. Liefländer et al. (2013) reported a marked difference in baseline nature connection levels between younger (9–10-year-old) and older (11–13-year-old) pupils. While both groups showed an increase in levels immediately post intervention, only the younger group (9–10) sustained this at the four-week follow up, indicating perhaps that changes in nature connection in younger children are more likely to be permanent.

### Length, Type of Intervention, and Environment

In studies that compared similar interventions with differing lengths, the longer interventions seemed to have a greater impact on nature connection (e.g., Sellmann and Bogner, 2013; Braun and Dierkes, 2017). As this trend is observed only within studies, it is impossible to determine whether it is the type, density, or length of the activity that has the effect.

**FIGURE 1:**
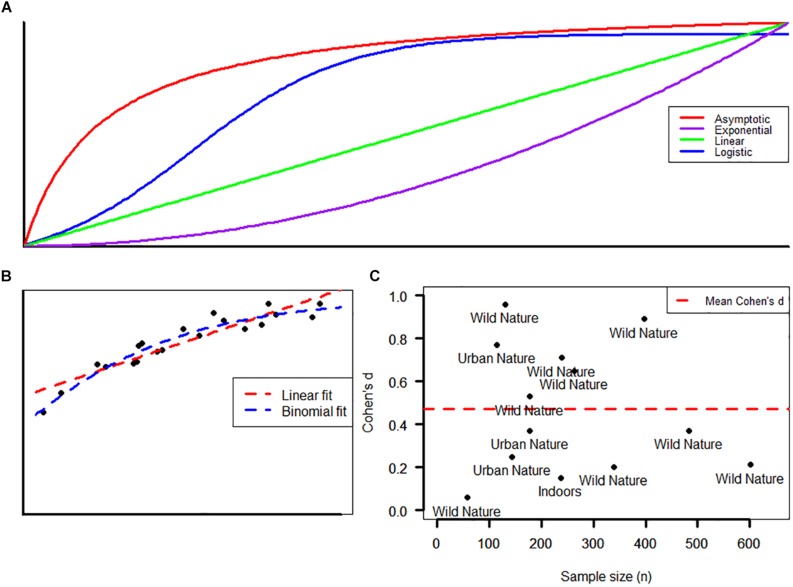
**(A)** Non-linear responses to an intervention. **(B)** A poorly fitted linear model and a better fit logistic/binomial model to a simulated curved dataset. **(C)** Standardized effect with increasing sample size, environment type as a text label.

Most of the activities reported on in the studies included in this review were knowledge-rich, with a distinct EE element. Kossack and Bogner (2012) report a negative effect of high information content, while Collado et al. (2013) suggest that enjoyment and play may have a positive effect on nature connection in children. Bruni et al. (2017) found that only the activity in which children engaged artistically with the natural world, such as narrative writing, art work, and photography, created a positive shift in nature connection. Immersive experiences and free outdoor play were seen as a positive feature by Mullenbach et al. (2018) as well as Schneider and Schaal (2017). There was heterogeneity in the types of environments reported, and these environments were idiosyncratic to the location of the study. As such it is difficult to draw any conclusions. A breakdown of different environments can be seen in Table 1, and in relation to effect size of intervention in Figure 1C.

### Baseline Connection to Nature and Ceiling Effect

Overall participants with lower nature connection during pre-testing seemed to make the biggest gain in most studies (Braun and Dierkes, 2017; Schneider and Schaal, 2017; Bruni et al., 2018; Boeve-de Pauw et al., 2019). This could be attributed to a number of potential factors, including a larger observed effect on children who had not previously had contact with natural environments, due to novelty. Additionally, this could be seen to support the Biophila hypothesis (Kellert and Wilson, 1995) that describes an innate tendency of humans to seek connection to nature. Potential methodological reasons may relate to the ceiling effect, explored below. Ceiling effects were reported in several studies (Ernst and Theimer, 2011; Kossack and Bogner, 2012; Barton et al., 2016) which may present a limitation of the instruments used to measure nature connection in young people, with the instrument technically only capable of measuring variation across 50% of its range of values. It may also be an interesting general property shared across many participants (i.e. an innate, above-average connection to nature).

### Analytic Issues and Implications Study Design

The response of the measure to an intervention may not be linear in nature (Figure 1A). For example, it may be that a hypothetical response to an intervention could rise quickly to a set level (asymptotic); have a threshold value resulting in a sharp increase to a leveling off point (logistic); have a constant rate of increase (exponential); or even in some rare cases the response could be linear. There is some tantalizing evidence that such non-linear relationships may exist, particularly when contrasting the effect of interventions in children at either end of the age range, or who start with different baseline nature connectedness scores (Braun and Dierkes, 2017).

Measurements from all but one of the instruments (IAT) have a second property that confounds their analysis using simple statistical tests. Variables from these instruments tend to be bounded (e.g., between one and five) with a great opportunity for variance at the center of a scale, and none at either extreme value. These mean–variance relationships tend not to conform well with linear regression, *t*-testing, and analysis of variance (ANOVA). This has been recognized by statisticians for some time (Nelder and Wedderburn, 1972) resulting in the development of generalized linear models (GLMs). Such models have matured with statistical computing and can explore the effect of independent variables and covariates on a plethora of measured outcomes. Beta, binomial, or quasibinomial GLMs are better suited to handling instruments with bounded outcomes, particularly when a ceiling or floor effect may be present (Figure 1B), and as such study design should take this into account to avoid poorly fitted models (Furr, 2011).

### Recommendations for Further Research

Using CEBM guidelines to evaluate evidence, we noted that most papers would be classified as level 3, i.e., non-randomized comparisons, with a single level 2 study, i.e., a RCT (Braun and Dierkes, 2017) and several level 4 studies, i.e., case series, or pre- and post-studies (Howick et al., 2011). We discuss our evaluation below, ending with recommendations for the field to move forward. There is an aphorism in science of “no controls, no conclusions” (Crawley, 2014, p. 8). Five of the 14 studies incorporated a form of control, with even fewer contrasting their intervention with that of a control set. While pre–post measurements do mitigate this issue somewhat, it is still impossible to discount the possibility of a confounding variable running alongside the intervention, inducing the change (Pearl, 2009). A creative approach would be to incorporate a wait-list control. Recruitment should employ some element of randomization (including cluster randomization) to remove the possibility of systematic confounding variables.

Sample sizes varied over two orders of magnitude (*n* = 58 to *n* = 601) and it is conspicuous that the largest studies also reported some of the smallest effects (Figure 1C). Large effects in underpowered experiments are common, due to the conflated false discovery rate (Friston, 2012). Related to this, statistical power (1−β) as estimated through a *post hoc* power analysis (Cohen, 1988) revealed a range of values from the lowest of 0.06 through to the highest of 1.0. This may point toward a likelihood of false negatives in the literature, though it should be noted that half of the studies generally met the conventional threshold of statistical power equaling 0.8 for hypothesis tests. In order to protect against false negatives, we suggest the following as a general guide for minimum sample size, based on effect sizes observed in the most robustly conducted piece of work (Braun and Dierkes, 2017). Assuming an effect size of Cohen’s *d* ≈ 0.2 a sample size of *n* = 400 for unpaired, and *n* = 200 for paired (pre–post) comparisons should be able to detect an effect.

We noted the array of statistical approaches employed throughout the literature, from a simple comparison of means (with no standard deviations) through to thorough mixed-model analysis of co-variance (ANCOVA). Two of the 14 employed omnibus tests with *post hoc* pairwise comparisons, the remainder conducted multiple pairwise comparisons without some form of correction to minimize the multiple comparisons problem. The problem in its simplest form is that every pairwise comparison carries a type I error rate for *m* hypotheses (α = 1−0.95^*m*^). A single comparison yields a rate of 0.05, five comparisons is 0.23, and 10 comparisons is 0.4. In this set of studies, the most extreme example found conducted 63 pairwise comparisons across a single dataset, yielding an α of 0.96 and meaning that there almost certainly would be false positive observations. This inflation of error can be corrected to mitigate this issue somewhat, through a variety of approaches, the simplest of which being the Bonferroni correction (Dunnett, 1955; Aickin and Gensler, 1996).

To that end we suggest that as a minimum, researchers should clearly report means and standard deviations for each level or group in their study; and for summary statistics a minimum of test statistic, degrees of freedom, *p*-values, and effect sizes. Where the raw data of the experiment require extensive manipulation it is advised to make the dataset publicly available in an anonymized fashion.

General guidelines from the open science framework (OSF) could be used to improve the reliability, reproducibility, and generalizability of studies in this field of environmental and educational psychology (Munafò et al., 2017). We have covered design and analysis above, but other cultural practices could be adopted, such as pre-registration (van’t Veer and Giner-Sorolla, 2016), reporting of null results and more transparency in the sharing of data and the analytical workflow.

## Conclusion

Throughout this review of studies that evaluate nature connection before and after different interventions, there is a notable absence of evaluations of different type of programs, for example nature kindergartens, forest schools, etc. An exception to this is the study by McCree et al. (2018) which evaluates several aspects of a forest school program in younger children. Part of the difficulty in making such evaluations is the fact that the majority of participants in such programs tend to be younger children (Knight, 2013), while at the same time no self-report instrument to measure nature connection in the early years’ age group currently exists (Barrable, 2019b). Finally, the hypothesis of a “critical period” for nature connection could be put to the test in future experimental research.

The majority of studies presented in this review explore EE programs, within a school or other educational context. However, new research suggests that the way to connect to nature is not necessarily through knowledge, but through beauty, emotion, and sustained contact (Lumber et al., 2017). More emphasis could be placed on measuring alternative activities that bring children in sustained or condensed contact with nature, such as forest schools, nature kindergartens, adventure activities, and wildlife expeditions.

Further research could include more non-educational interventions that look at the interaction between play or mindfulness, and nature connection (such as ones focused on adults, see Unsworth et al., 2016). Finally, being clear about our intention to facilitate nature connection in children and differentiating between simply providing children with opportunities to be in nature and fostering and nurturing connectedness could further help to identify and highlight which activities are most suited to increasing a child’s connection to the natural world.

The review identifies some points of note: One relates to age, and is in accordance with previous literature that highlight the importance of early emotional connection to nature (Wells and Lekies, 2006; Jalongo, 2014). Moreover, earlier studies have found that length of time, as well as time spent in nature during childhood are the two most significant predictors of emotional affinity toward the natural world (Kals et al., 1999; Andrejewski et al., 2011). This review reinforces this and further highlights the fact that changes in nature connection in younger children may be more resistant to change over time.

The second point relates to the way we measure nature connection and possible limitations of our current instruments. This includes limitations in the age-range of validated measures, no self-report measures currently exist for children under 8 years of age (Barrable, 2019a), as well as the fact that current measures may impose an artificial ceiling effect that prevents us from measuring changes in highly connected individuals.

Finally, the last point raised in this review relates to the design, recruitment, and consistency of reporting, which makes the quality of the evidence weaker than it could be, given the amount of effort and relative ease with which they could be rectified. To that effect, we propose the above guidelines for future research and reporting in this field.

## Author Contributions

AB conceived, designed, and undertook the review. DB reviewed the search results, assisted in compiling the table, and undertook the statistical analysis, reporting, and visualizations. AB drafted the final report. AB and DB contributed and approved the final version of the manuscript.

## Conflict of Interest

The authors declare that the research was conducted in the absence of any commercial or financial relationships that could be construed as a potential conflict of interest.
